# Electrophysiological correlates of selective attention: A lifespan comparison

**DOI:** 10.1186/1471-2202-9-18

**Published:** 2008-01-31

**Authors:** Viktor Mueller, Yvonne Brehmer, Timo von Oertzen, Shu-Chen Li, Ulman Lindenberger

**Affiliations:** 1 School of Psychology, Saarland University, Im Stadtwald 1, 66123 Saarbrücken, Germany; 2 Center for Lifespan Psychology, Max Planck Institute for Human Development, Lentzeallee 94, 14195 Berlin, Germany

## Abstract

**Background:**

To study how event-related brain potentials (ERPs) and underlying cortical mechanisms of selective attention change from childhood to old age, we investigated lifespan age differences in ERPs during an auditory oddball task in four age groups including 24 younger children (9–10 years), 28 older children (11–12 years), 31 younger adults (18–25), and 28 older adults (63–74 years). In the Unattend condition, participants were asked to simply listen to the tones. In the Attend condition, participants were asked to count the deviant stimuli. Five primary ERP components (N1, P2, N2, P3 and N3) were extracted for deviant stimuli under Attend conditions for lifespan comparison. Furthermore, Mismatch Negativity (MMN) and Late Discriminative Negativity (LDN) were computed as difference waves between deviant and standard tones, whereas Early and Late Processing Negativity (EPN and LPN) were calculated as difference waves between tones processed under Attend and Unattend conditions. These four secondary ERP-derived measures were taken as indicators for change detection (MMN and LDN) and selective attention (EPN and LPN), respectively. To examine lifespan age differences, the derived difference-wave components for attended (MMN and LDN) and deviant (EPN and LPN) stimuli were specifically compared across the four age groups.

**Results:**

Both primary and secondary ERP components showed age-related differences in peak amplitude, peak latency, and topological distribution. The P2 amplitude was higher in adults compared to children, whereas N2 showed the opposite effect. P3 peak amplitude was higher in older children and younger adults than in older adults. The amplitudes of N3, LDN, and LPN were higher in older children compared with both of the adult groups. In addition, both P3 and N3 peak latencies were significantly longer in older than in younger adults. Interestingly, in the young adult sample P3 peak amplitude correlated positively and P3 peak latency correlated negatively with performance in the Identical Picture test, a marker measure of fluid intelligence.

**Conclusion:**

The present findings suggest that patterns of event-related brain potentials are highly malleable within individuals and undergo profound reorganization from childhood to adulthood and old age.

## Background

Scalp-recorded event-related brain potentials (ERPs) derived from electroencephalogram (EEG) play an important role in studies of cortical correlates of cognitive processes, primarily because of their relatively high temporal resolution (see [1,2] for reviews). The non-invasive nature of EEG assessments makes them particularly suitable for studying neurocognitive development in infants and children [3,4]. To date, age-comparative studies compared electrophysiological correlates of cognition either across childhood, adolescence and adulthood (e.g., [4-9]) or across adulthood and old age (e.g., [10-12]. Other than a few studies covering a wide age range of lifespan development [13,14], changes in brain electrophysiological activity have primarily been investigated with respect to either child development or aging. The aim of the present study was to explore changes in brain electrophysiological activity (also attention-related activity) across the lifespan.

ERPs reflect invariant changes of ongoing EEG activity evoked by the stimulus. ERP components are usually quantified by their peak amplitudes and peak latencies. The most prominent ERP components observed in studies of selective attention using the auditory oddball paradigm are N1, P2, N2 and P3, with peak latencies at about 100, 150, 200 and 300 ms after the stimulus onset, respectively. It is usually assumed that the N1 and P2 components reflect automatic stimulus processing that is influenced by early attention and orientation processes (e.g., [15]). The N1-P2 deflection has also been considered as an indicator for the cortical arousal response [16]. The N2 component is usually assumed to reflect the classification or categorization of deviant stimuli [17]. Finally, P3 is generally regarded as a more "cognitive", "endogenous," or "top-down" component that reflects context updating, orientation, processing termination, decision-making, and working memory (e.g., [1,18-20]). P3 peak latency has also been found to indicate speed of stimulus processing [21,22]. A further ERP component is the N3 or Slow Wave (SW: [14,23]). N3 may indicate enhanced attention to the stimulus, as it tends to be elicited in response to surprising, interesting, or important stimuli. In line with this interpretation, a similar late negativity was found when subjects had to redirect their attention back to a task after being distracted by novel environmental sounds [24,25] or after unexpected frequency changes in auditory stimuli [26,27]. N3 can be superimposed by CNV (Contingent Negative Variation), a slow negative component that in S1-S2 paradigms is related to fronto-centrally distributed negativity reflecting anticipation or expectancy [28,29]. Recently, the CNV was investigated in the modified oddball paradigm to compare preparatory and decision mechanisms and their sensitivity to variations in target probability [30]. CNV was not modulated by target probability with the exception that the CNV amplitude was low when target probability was zero. Below we review the effects of different ERP components assessed in the two-tone oddball-paradigm as a function of age.

Age differences in the waveform of the various ERP components between childhood and adulthood are inconsistent (see summary in Table 1). Several authors [31-34] indicated that the auditory *N1 *cannot be consistently elicited in children under the age of 8 or 9 years, and that it only becomes adult-like at about 16 years of age. Bruneau et al. [35] have shown maximal amplitude of the midtemporal responses in younger children (4–8 years) peaking at about 170 ms and fronto-central N1 maxima with a peak latency at about 100 ms in adults. In addition, Čeponiene et al. [36] reported protracted maturation of the N1 component, while the N2 component becomes increasingly robust during mid-childhood (3–6 years). Ladish and Polich [37] found an increase in N1 amplitude and a decrease in N1 latency with increasing age from 5 to 19 years. Johnstone et al. [8] found a linear decrease in N1 amplitude and latency for target tones from 8 to 17 years. Similarly, Fuchigami et al. [38] showed that N1 peak latency became progressively shorter in children from 4 to 16–17 years and reached adult levels thereafter. In contrast, Johnson [39] could not find significant changes in N1 (nor in P2 and N2) peak amplitude and peak latency for auditory modality between 7 and 20 years of age. Similarly, Goodin et al. [13] also found similar N1 latency between children (6 to 15 years) and young adults. As for findings regarding adult development and aging [10], N1 amplitude increased significantly with age and increased N1 latency was only significant in the posterior region.

**Table 1:** Age-related changes in peak amplitude and peak latency of different ERP components in different studies

Study	N1	P2	N2	P3	N3
	Peak amplitude				
[38]	n.s.		n.s.	n.s.	-
[43]				8–13 yrs. ↓	8–13 yrs. ↓
[39]	7–20 yrs. n.s.	7–20 yrs. n.s.	7–20 yrs. n.s.	7–20 yrs. ↓	-
[8]	8–17 yrs. ↓	8–17 yrs. ↑	8–17 yrs. ↓	8–17 yrs. ↑	-
[37]	5–19 yrs. ↑	5–19 yrs. ↑	5–19 yrs. ↓	5–19 yrs. ↑	-
[13]	6–15 yrs. n.s. 15–76 yrs. ↓	6–15 yrs. n.s. 15–76 yrs. ↓	6–15 yrs. n.s. 15–76 yrs. ↓	6–15 yrs. n.s. 15–76 yrs. ↓	-
[10]	20–89 yrs. ↑	20–60 yrs. ↑, 60–89 yrs. ↓	20–89 yrs. ↓ only fronto- central	20–89 yrs. ↓	-
[78]	22.5 vs. 78.6 yrs. n.s.	22.5 vs. 78.6 yrs. ↑		22.5 vs. 78.6 yrs. n.s.	22.5 vs. 78.6 yrs. ↓
[11]	18–90 yrs. ↑	18–90 yrs. ↑	18–90 yrs. n.s.	18–90 yrs. ↓	18–90 yrs. ↓
[14]	20–79 yrs. n.s.	20–79 yrs. n.s.	20–79 yrs. n.s.	20–79 yrs. ↓ 0.18 mV/year	20–79 yrs. ↓ 0.05 mV/year
[79]	20.3 vs. 66.6 yrs. n.s.	20.3 vs. 66.6 yrs. ↑	20.3 vs. 66.6 yrs. ↓	20.3 vs. 66.6 yrs. ↓	
[42]				21–34 yrs. ↓ 35–64 yrs. ↑ 65–94 yrs. ↓	
	Peak Latency				
[38]	4–16/17 yrs. ↓, thereafter adult level		4–16/17 yrs. ↓, thereafter adult level	4–16/17 yrs. ↓, thereafter adult level	-
[43]				8–13 yrs. n.s.	-
[39]	7–20 yrs. n.s.	7–20 yrs. n.s.	7–20 yrs. n.s.	7–20 yrs. ↓	-
[8]	8–17 yrs. ↓	8–17 yrs. n.s.	8–17 yrs. ↓	8–17 yrs ↓	-
[37]	5–19 yrs. ↓	5–19 yrs. ↓	5–19 yrs. ↓	5–19 yrs. ↓	-
[13]	6–76 yrs. ↑	15–76 yrs. ↑ 0.7 ms/year	6–15 yrs. ↓ 12.3 ms/year 16–76 yrs. ↑ 0.8 ms/year	6–15 yrs. ↓ 18.4 ms/year 16–76 yrs. ↑ 1,8 ms/year	-
[10]	20–89 yrs. ↑ only posterior	20–60 yrs. ↑ only anterior	20–89 yrs. ↑	Up to 60 yrs. ↑	-
[78]	22.5 vs. 78.6 yrs. n.s.	22.5 vs. 78.6 yrs. ↑		22.5 vs. 78.6 yrs. ↑	-
[11]	18–90 yrs. n.s.	18–90 yrs. n.s.	18–90 yrs. ↑	18–90 yrs. ↑	-
[14]	20–79 yrs. n.s.	20–79 yrs. ↑ 0.25 ms/year	20–79 yrs. ↑ 0.65 ms/year	20–79 yrs. ↑ 1.36 ms/year	20–79 yrs. ↑ 0.89 ms/year
[79]	20.3 vs. 66.6 yrs. n.s.	20.3 vs. 66.6 yrs. n.s.	20.3 vs. 66.6 yrs. ↑	20.3 vs. 66.6 yrs. ↑	
[42]				21–94 yrs. ↑	

↑ = Increase, ↓ = Decrease, n.s. = no significant changes with age

In the study discussed above [8], *P2 *amplitude displayed a linear increase from 8 to17 years, whereas P2 latency was similar across these age groups. With respect to adulthood and old age, Anderer et al. [10] found that P2 amplitude increased from 20 to 60 years and decreased thereafter; P2 latency increased with advancing age, but only in anterior regions. In a lifespan study covering the age range from 15 to 76 years of age [13], the latency of P2 increased significantly with age at a rate of about 0.7 ms per year. Similarly, in the study by Picton et al. [14], P2 peak latency also increased significantly with age from 20 to 79 years, but at a rate of about 0.25 ms per year. Neither of the two studies observed P2 amplitude differences as a function of adult age.

With respect to changes of the *N2 *component from childhood to adulthood, most studies [8,37,38] showed a decrease in the N2 amplitude and latency. Similarly, Goodin et al. [13] found a decrease in N2 latency with age in children of 6 to 15 years but an increase in this latency after the age of 15. With respect to adulthood, Picton et al. [14] reported a significant increase in N2 peak latency with age at a rate of 0.65 ms per year in adults of 20 to 79 years (for similar results, see [10]).

In the transition from childhood to young adulthood, changes in *P3 *primarily consist in increasing peak amplitude and decreasing peak latency [8]. Ladish and Polich [37] found an increase in P3 amplitude at centro-parietal sites and an overall decrease in P3 latency with increasing age from 5 to 19 years. Overall, the decrease in P300 peak latency during child development is a common finding [38-40]. Goodin et al. [13] observed a significant decrease with age in the latency of P3 at a rate of 18.4 ms/year in children between 6 and 15 years, while the opposite effect (i.e. a significant increase of P3 latency with age at a rate of 1.8 ms/year) was found in adults between 15 and 76 years of age. In addition, the peak-to-peak N2-P3 amplitude in children (6–15 years) showed no significant changes with age, whereas adult N2-P3 amplitude decreased significantly with age (from 15 to 76 years). Contrary to the development during childhood, adulthood is mostly associated with decreasing P3 amplitude and increasing P3 peak latency [10,11,13,14,41,42].

With respect to the *N3 *component, Gumenyuk et al. [43] showed significantly greater Late Negativity (LN that is similar to N3) amplitude across the frontal sites in young children (8–9 years) than children in mid (10–11 years) and late (12–13 years) childhood. Picton et al. [14] distinguished two SW (or N3) components: frontal and parietal. Frontal negative SW displayed a significant decrease in peak amplitude and a significant increase in peak latency with age throughout adulthood. Interestingly, the maturational time course of Nc (Negative Component, also similar to N3), i.e., an increase in amplitude across infancy and early childhood followed by a gradual decline through adolescence, was noted to closely parallel synaptic density changes in the frontal cortex [44].

In sum, most findings suggest that the amplitude of ERP components increases from childhood to adulthood and decreases thereafter, whereas the peak latency decreases from childhood to adulthood and increases thereafter. The N2 seems to form an exception to this empirical regularity because both its amplitude and latency decrease from middle childhood to old age, perhaps reflecting the relatively early maturation of this component [36].

In addition to the standard ERP components commonly found with the oddball task mentioned above, various difference waves – derived from taking the difference between responses to standard and deviant stimuli or between responses made under Attend and Unattend conditions – are typically used as indicators of different aspects of stimulus change detection and attentional processes. For instance, an earlier and a later negative difference wave can be derived by taking the differences between responses to deviant and standard tones, known as "Mismatch Negativity" (MMN) and "Late Discriminative Negativity" (LDN), respectively. MMN is an indicator of stimulus change or of a neural-mismatch process triggered by the sensory input from a rare deviant stimulus at the presence of a neural trace of the frequent standard stimulus [45,46]. This process is seen as an automatic, preconscious change-detection mechanism [46] or as the operation of a permanent feature-detector system [47]. The LDN might reflect certain aspects of sound discrimination, since it is elicited in an oddball paradigm in response to deviant sounds. Näätänen et al. [48] have suggested that in adults, such later activity might be associated with "sensitisation processes" after a stimulus change and may serve as an automatic preparatory process for the detection of any additional changes. However, the functional role of LDN in information processing and its age-related changes are far from clear.

Since the *MMN *has been observed in children and infants, including pre-term newborns [49], and even in the human fetal brain [50], it appears to reflect information-processing mechanisms installed very early during ontogenesis. At the same time, several studies have shown significant age-associated differences in the amplitude and latency of MMN [51-55]. Some studies reported a slight MMN peak latency decrease during the school-age years [55,56] and somewhat greater MMN amplitude in school-age children than in adults [57]. In adults, MMN has been shown to have a fronto-central scalp distribution [58] that is more central in children [51]. Older adults have been found to have smaller MMN amplitude than young adults in some studies [52,59] while in other studies, young and older adults displayed similar MMN amplitudes [60]. Pekkonen et al. [61] observed reduced MMN amplitude in older adults when the stimuli were presented at long inter-stimulus intervals (e.g., 3 seconds), whereas no age-related reduction in MMN amplitude was observed when the stimuli were presented at relatively short inter-stimulus intervals (e.g., 1 second). Several differences between the two negative responses (i.e., MMN and LDN) are apparent in terms of developmental changes. MMN is developmentally quite stable. Although *LDN *can also be found in newborns and the fetal brain [50], however, it can be observed most reliably in young children, and its amplitude decreases as a function of age [62,63].

"Processing Negativity" (PN) or "Negative difference wave" (Nd) represents another important ERP difference wave component [64,65]. The terms PN and Nd are often used as synonymous reflecting the difference between ERPs to the same stimulus when attended and when not attended. According to Näätänen and collegues [45,66], PN reflects a comparison process between a stimulus and the attentional trace. Subtracting the ERP to the unattended stimulus from that to the attended stimulus yields only the PN differential. The term PN used in our study is related to this PN differential and is like Nd a difference wave between ERPs for attended and unattended stimuli. PN is related to some form of extra processing assigned to attended events on the basis of a preceding selection process. Thus, it is an indicator of voluntary selective attention. Although PN has been more often determined in dichotic listening (two-channels) studies and mostly for standard stimuli, there is evidence suggesting that this difference wave component can also be identified in the standard one-channel oddball paradigm for both standard and deviant stimuli [65,67]. Here we distinguish between early and late PN components measured in the one-channel oddball paradigm, both with fronto-central maxima. The early component is proposed to reflect the processing of the sensory stimulus features and the later component to reflect further processing of the stimuli and rehearsal of the attentional trace [68,69]. Developmental and aging studies of PN are very sparse and have provided mixed results. Berman and Friedman [70] found an increase in early Nd (or PN) amplitude and a decrease in its latency from childhood (mean age 8.1) to adulthood (mean age 23.8). Bartgis et al. [71] showed a significant increase in Nd amplitude in children from 5 to 9 years. In a study with 9 and 12 year-old children as well as adults, Gomes et al. [72] found a significantly longer Nd peak latency in children as compared with adults, but no age differences in Nd peak amplitude.

Empirical data suggest that although the subcortical auditory pathway shows very rapid maturation, auditory stimulus processing on the cortical level protracts its maturation into adolescence [35,73]. At the neural level, anatomical, chemical, and functional evidence suggests that prefrontal cortex and associated neural networks undergo profound age-based changes well into adolescence. Specifically, the maturational gradients of PFC (prefrontal cortex) and ACC (anterior cingulate cortex) are very protracted, with continuing development until early adulthood (see for review [74]. The effects of aging on PFC and ACC are also well established and pronounced [75-77].

As reviewed above, there is a lack of lifespan studies that directly compare electrophysiological correlates of stimulus processing and selective attention across childhood, adulthood, and old age. The inconsistencies of findings across studies examining only a portion of the lifespan (as summarized in Table 1) in part could result from the differences in the experimental paradigms used. Applying exactly the same experimental paradigm across four age groups covering the lifespan, the goal of this study was to more directly examine lifespan age differences in electrophysiological correlates of selective attention mechanisms with respect to the various primary ERP components and derived different difference waves. Age-related changes in peak amplitude and peak latency of ERPs and their direction can be obtained to some extent from the literature reviewed above. More specifically, we expect that negative waves, above all N2 and N3, reflecting automatic and non-automatic activation during the task, as well as derived from these components difference waves (MMN, LDN and PN), reflecting changes in stimulus detection and selective attention, will be higher in children than in adults. P3 wave affected by memory driven energy resource allocation could be critically for adult age changes or aging. In this connection, we expect reduced P3 peak amplitude and prolonged P3 peak latency in older adults as compared with other age groups. Besides the age-related changes in peak amplitude and peak latency, we expect also changes in topological distribution of corresponding ERPs, which will be more pronounced in antero-posterior axis, related to developmental and also aging-related changes in frontal and also parietal brain regions. However, age-related changes in the lateral axis could also be expected [10,35,54,64].

## Results

### Event-related potential components

#### Waveforms and topological distributions

**Figure 1:**
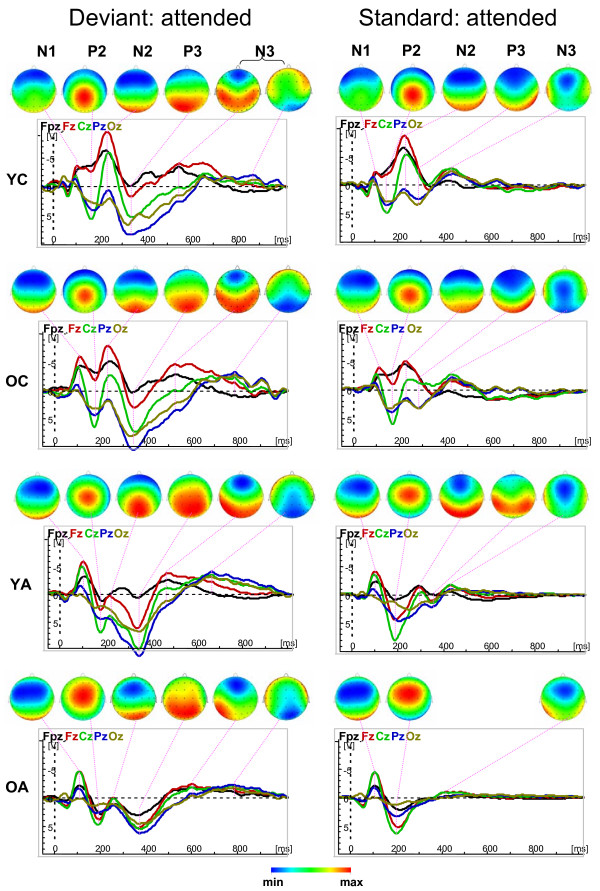
Waveforms of the five midline electrodes (Fpz, Fz, Cz, Pz and Oz) and the topological distribution of the ERP components (N1, P2, N2, P3, and N3) for deviant (left column) and standard (right column) stimuli under Attend condition across ages (YC = Younger Children, OC = Older Children, YA = Younger Adults, OA = Older Adults). Note that the brain maps have different scaling and that the N3 wave for deviant stimuli contains two subcomponents with frontal and parieto-occipital maxima.

**Figure 2:**
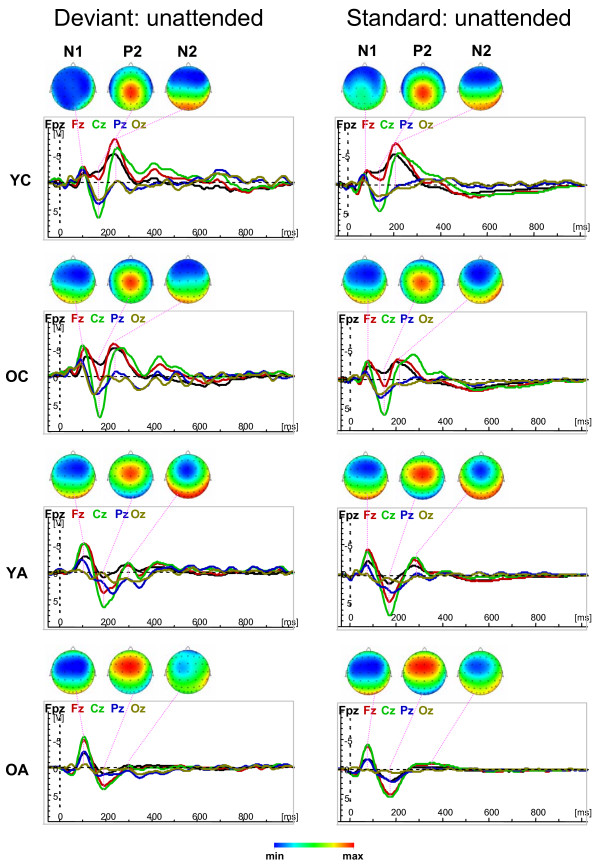
Waveforms of the five midline electrodes (Fpz, Fz, Cz, Pz and Oz) and the topological distribution of the ERP components (N1, P2, and N2) for deviant (left column) and standard (right column) stimuli under Unattend condition across ages (YC = Younger Children, OC = Older Children, YA = Younger Adults, OA = Older Adults). Note that under Unattend condition ERPs are reduced to three early components (N1, P2, and N2) and the later components (P3 and N3) appear in a redundant form or failed. Note also that the brain maps have different scaling.

Fig. 1 and 2 show the waveforms of the five midline electrodes (Fpz, Fz, Cz, Pz and Oz) and the topological distribution of the ERP components under the Attend and Unattend conditions for deviant and standard stimuli across age groups. Results show that the maximal amplitude of the N1 component is frontally localized in young children and shifts to the central sites with advancing age, whereas the P2 maximum is localized in the parietal regions in young children and shifts towards the frontal regions with advancing age. The N2 component shows a frontal maximum that is less localizable in the young and older adults because this component is substantially reduced and is mostly positive in adults compared with children. Older adults showed N2 wave only for attended deviant stimuli; in the other conditions their ERPs are reduced by up to two components, e.g., N1 and P2. The P3 and N3 components are evident under Attend conditions for deviant stimulus but are markedly reduced for the standard stimulus under Attend conditions and for both stimuli (deviant and standard) under Unattend conditions. With attended deviant stimuli, P3 amplitude is more pronounced in children's parieto-occipital region and also shows central maximum, especially in young adults. The N3 component shows practically two peaks with an earlier maximum frontally and a later maximum in the parieto-occipital region. The parieto-occipital maximum of N3 in young children is considerably reduced compared with all other age groups. Statistical analysis for peak amplitude and peak latency was carried out for attended deviant only, because comparisons across ages for other conditions are complicated due to the varied structures of the different ERP components. However, lifespan age differences in change detection and selective attention mechanisms could be observed with respect to difference wave measures as described below.

#### Peak amplitude

**Figure 3:**
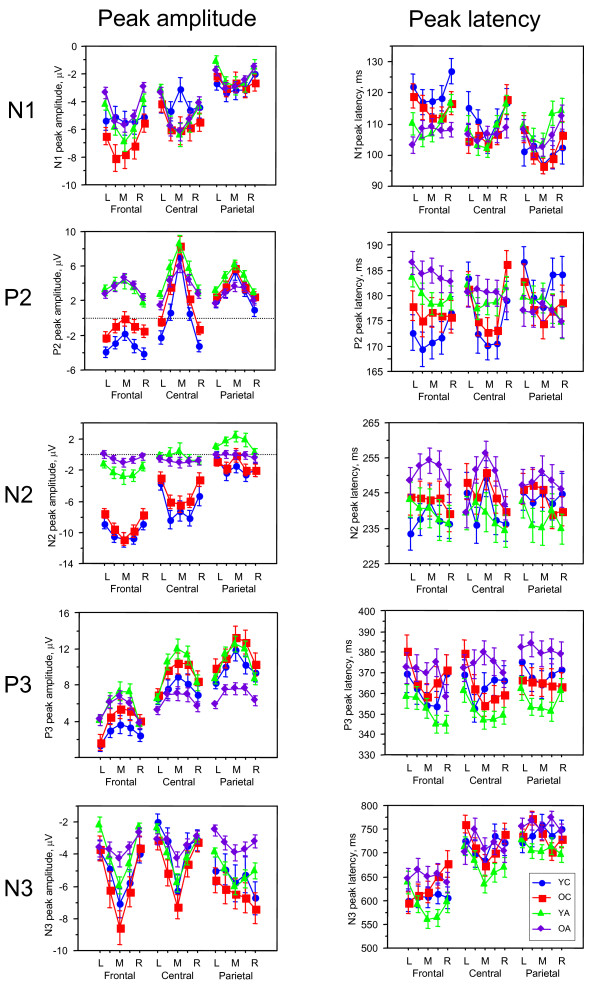
Diagrams for mean peak amplitude (left column) and mean peak latency (right column) of the 5 ERP components (N1, P2, N2, P3, and N3) depicted for 5 frontal, 5 central and 5 parietal sites: left (L), medium left, mid-sagittal (M), medium right, and right (R). Age groups are coded with different symbols (s. legend at the bottom right).

For deviant stimulus in the Attend condition, a three-way repeated measures ANOVA (Age × Antero-Posterior × Laterality) with peak amplitude as a dependent variable revealed a significant main effect of the factor Age (except N1) and significant interactions Age × Antero-Posterior (except N3), Age × Laterality, and Age × Antero-Posterior × Laterality for all ERP components. The ANOVA results data is presented in Table 2. A post-hoc Fischer's PLSD test showed that P2 amplitude was higher in adults than in children, and higher in the OC than in the YC (YA, OA > OC > YC). In contrast, children had a higher N2 amplitude than adults (YC, OC > YA, OA). P3 amplitude was lower in OA compared with YA and OC (YA, OC > OA). A post-hoc Fischer's PLSD test for N3 amplitude also showed it to be higher in older children compared with both adult groups (OC > YA, OA). Significant interactions of the factor Age with the factors Laterality and Antero-Posterior indicated that the topological distribution of these ERP components alters with age (for details s. Fig. 3).

**Table 2:** ANOVA results (*F *and *p *values) for peak amplitude and peak latency of the five ERP components for the deviant stimuli in the Attend conditions

Components	Effects			
	Age (df = 3,107)	Age × Antero-Posterior (df = 6,412)	Age × Laterality (df = 12,428)	Age × Antero-Posterior × Laterality (df = 24,856)
Peak Amplitude				
N1	1.1 (n.s.)	3.4 (*p *= 0.01)	29.6 (*p *< 0.0001)	2.1 (*p *= 0.005)
P2	12.8 (*p *< 0.0001)	19.7 (*p *< 0.0001)	3.9 (*p *< 0.0001)	5.3 (*p *< 0.0001)
N2	26.8 (*p *< 0.0001)	29.5 (*p *< 0.0001)	4.1 (*p *< 0.0001)	3.0 (*p *< 0.0001)
P3	2.9 (*p *= 0.04)	11.5 (*p *< 0.0001)	2.6 (*p *= 0.01)	2.0 (*p *= 0.009)
N3	3.6 (*p *= 0.02)	1.8 (n.s.)	2.9 (*p *= 0.004)	3.8 (*p *< 0.0001)
Peak Latency				
N1	0.6 (n.s.)	7.2 (*p *< 0.0001)	1.1 (n.s.)	1.2 (n.s.)
P2	0.8 (n.s.)	5.8 (*p *< 0.0001)	1.8 (n.s.)	1.4 (n.s.)
N2	1.7 (n.s.)	1.0 (n.s.)	1.4 (n.s.)	0.9 (n.s.)
P3	4.0 (*p *= 0.01)	1.2 (n.s.)	2.2 (*p *= 0.022)	1.6 (*p *= 0.05)
N3	2.9 (*p *= 0.04)	0.5 (n.s.)	2.3 (*p *= 0.018)	2.1 (*p *= 0.003)

n.s. = not significant

#### Peak latency

A statistical analysis of ERPs latency revealed a significant main effect of the factor Age for the P3 and N3 components, and significant interactions Age × Laterality for P3 and N3, Age × Antero-Posterior for N1 and P2, and Age × Laterality × Antero-Posterior for the P3 and N3 ERP components. The ANOVA results data is presented in Table 2. A post-hoc Fischer's PLSD test showed that P3 and N3 peak latency was significantly prolonged in older adults than in younger adults (OA > YA), and N3 peak latency was, furthermore, significantly shorter in YA compared with OC (OC > YA: s. Fig. 3 for details).

### ERP difference wave components for stimulus change

**Figure 4:**
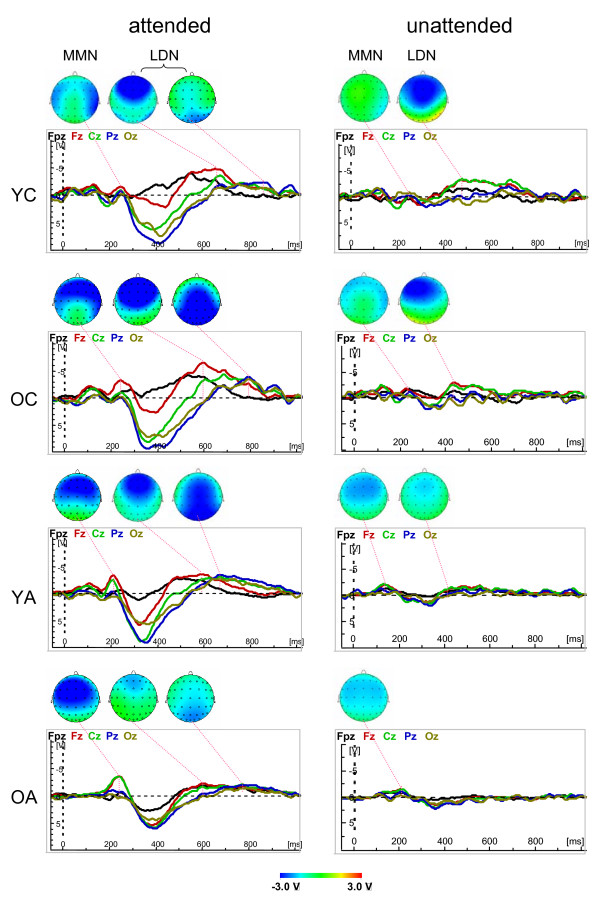
Waveforms of the five midline electrodes (Fpz, Fz, Cz, Pz and Oz) and the topological distribution of the difference wave components reflecting stimulus change detection (MMN and LDN) for attended (left column) and unattended (right column) stimuli across ages (YC = Younger Children, OC = Older Children, YA = Younger Adults, OA = Older Adults). Note that the LDN wave for attended stimuli contains two subcomponents with frontal and parieto-occipital maxima.

Figure 4 shows the ERP difference waves between deviant and standard stimuli (MMN and LDN) of the five midline electrodes (Fpz, Fz, Cz, Pz and Oz) and the topological distribution of these waveforms in the Attend and Unattend conditions across age groups. It is evident that MMN and LDN are substantially reduced in Unattend relative to the Attend condition, especially in older and younger adults. It is also seen that MMN, especially under Attend conditions, has two subcomponents: an earlier peak at about 100–120 ms and a later peak at about 220–240 ms. For the present purpose of lifespan comparisons, we analyzed the second component only (time window 150–300 ms), given that the earlier component was absent in older adults. In the Attend condition, the maximal amplitude of the MMN was temporally more localized in younger children and shifts to the fronto-central sites with advancing age. Like N3, LDN has two peaks: an earlier maximum frontally and a later parieto-occipital maximum. The parieto-occipital maximum in young children is also (like the N3 component) considerably reduced compared with older children and younger adults. In older adults, both of the LDN components are reduced. On the basis that the difference wave components are considerably reduced in the Unattend condition so that age-related comparison is not feasible, statistical analyses were restricted to the Attend condition.

**Figure 5:**
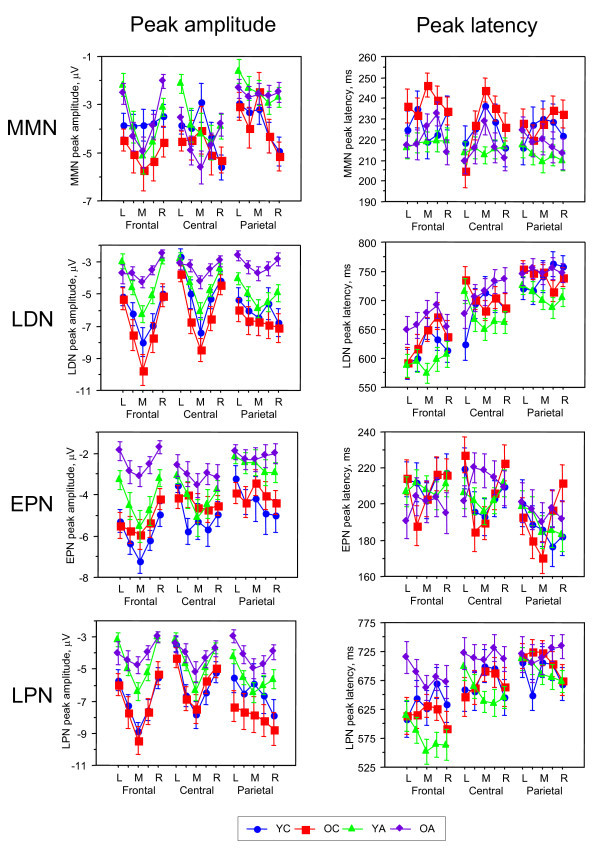
Diagrams for mean peak amplitude (left column) and mean peak latency (right column) of the 4 difference wave components (MMN, LDN, EPN, and LPN) depicted for 5 frontal, 5 central and 5 parietal sites: left (L), medium left, mid-sagittal (M), medium right, and right (R). Age groups are coded with different symbols (s. legend at the bottom).

A three-way repeated measures ANOVA (Age × Laterality × Antero-Posterior) with peak amplitude as a dependent variable revealed a significant main effect of the factor Age for LDN and significant interactions Age × Laterality and Age × Laterality × Antero-Posterior for both MMN and LDN, and a significant interaction Age × Antero-Posterior for the MMN-amplitude only. The ANOVA results data is presented in Table 3. A post-hoc Fischer's PLSD test showed that LDN amplitude was higher in the YC compared with the OA (YC > OA), and in the OC higher than in both YA and OA (OC > YA, OA). Significant interactions of the factor Age with the factors Laterality and Antero-Posterior indicated that the topological distribution of MMN and LDN alters with age (for details s. Fig. 5).

**Table 3:** ANOVA results (*F *and *p *values) for peak amplitude and peak latency of the ERP difference wave components in the Attend conditions

Components	Effects			
	Age (df = 3,107)	Age × Antero-Posterior (df = 6,412)	Age × Laterality (df = 12,428)	Age × Antero-Posterior × Laterality (df = 24,856)
Peak Amplitude				
MMN	1.1 (n.s.)	2.6 (*p *= 0.02)	4.9 (*p *< 0.0001)	2.4 (*p *= 0.002)
LDN	9.8 (*p *< 0.0001)	1.2 (n.s.)	2.9 (*p *= 0.003)	3.4 (*p *< 0.0001)
EPN	5.8 (*p *= 0.001)	2.4 (*p *= 0.05)	1.2 (n.s.)	1.0 (n.s.)
LPN	7.1 (*p *< 0.0001)	2.0 (n.s.)	1.1 (n.s.)	3.0 (*p *< 0.0001)
Peak Latency				
MMN	2.8 (*p *= 0.04)	0.4 (n.s.)	1.3 (n.s.)	1.7 (*p *= 0.027)
LDN	3.0 (*p *= 0.03)	0.7 (n.s.)	2.8 (*p *= 0.002)	1.9 (*p *= 0.009)
EPN	0.0 (n.s.)	1.1 (n.s.)	3.3 (*p *= 0.001)	1.1 (n.s.)
LPN	3.5 (*p *= 0.02)	1.9 (n.s.)	1.9 (n.s.)	0.7 (n.s.)

n.s. = not significant

A statistical analysis for MMN and LDN latency revealed a significant main effect of the factor Age on both components, as well as significant interactions Age × Laterality for LDN latency, and Age × Laterality × Antero-Posterior also for both difference waves components (s. Table 3 and Fig. 5). A post-hoc Fischer's PLSD test showed prolonged MMN-latency in OC compared with both adult groups (OC > YA, OA), and prolonged LDN latency in OC compared with YA (OC > YA). There was also prolonged LDN latency in OA compared with YA (OA > YA).

### ERP difference wave components for selective attention

**Figure 6:**
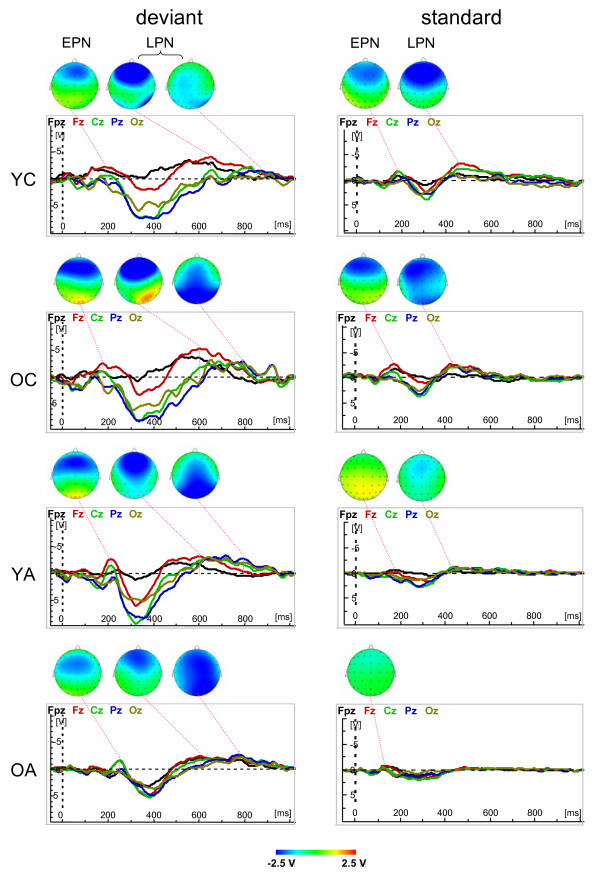
Waveforms of the five midline electrodes (Fpz, Fz, Cz, Pz and Oz) and the topological distribution of the difference wave components reflecting selective attention (EPN and LPN) for deviant (left column) and standard (right column) stimuli across ages (YC = Younger Children, OC = Older Children, YA = Younger Adults, OA = Older Adults). Note that the LPN wave for deviant stimuli contains two subcomponents with frontal and parieto-occipital maxima.

Figure 6 shows the ERP difference waves between attend and unattend conditions (EPN and LPN) of the five midline electrodes (Fpz, Fz, Cz, Pz and Oz) and the topological distribution of these waveforms for deviant and standard stimuli across age groups. For deviant stimuli, it shows that the earlier PN (EPN) peaks at 200 ms and, like N2, has a fronto-central distribution. LPN has, like N3, two peaks: an earlier frontal maximum and a later parieto-occipital maximum. In older adults, the frontal LPN component begins earlier than the parieto-occipital LPN component but goes down later, practically at the time of the parieto-occipital maximum, so that the frontal maximum could also be seen at the time of the parieto-occipital maximum. For standard stimuli, both difference wave components (EPN and LPN) are considerably reduced, especially for younger and older adults. Therefore, subsequent statistical analyses were conducted only with respect to deviant stimulus.

A three-way repeated measures ANOVA (Age × Laterality × Antero-Posterior) with peak amplitude as a dependent variable revealed a significant main effect of the factor Age for both early and late PN and significant interactions Age × Antero-Posterior for EPN and Age × Laterality × Antero-Posterior for LPN. The ANOVA results data is presented in Table 3. A post-hoc Fischer's PLSD test showed that EPN amplitude was greater in the YC compared with the YA and OA (YC > YA, OA), and greater in the OC than in the OA (OC > OA). LPN amplitude was higher in children than in adults (YC, OC > YA, OA). See Fig. 5 for details on the differences between the age groups in the topological distribution. It is evident that these differences in EPN are localized above all frontally, and in LPN at the frontal and lateral parietal sites.

A statistical analysis for EPN and LPN latency revealed a significant main effect of the factor Age for LPN, and a significant interaction Age × Laterality for EPN latency (s. Table 3). A post-hoc Fischer's PLSD test showed prolonged LPN latency in OA compared with OC and YA (OA > OC, YA).

### Correlations between behavioral data and ERP components

**Figure 7:**
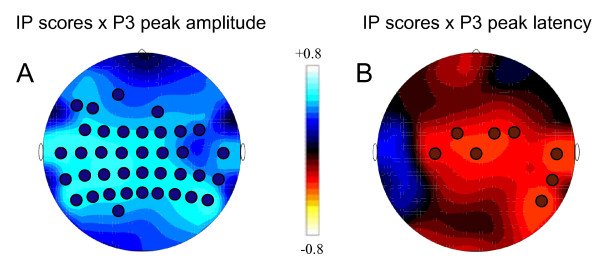
Brain maps for young adults with correlation coefficient distributions. Panel A displays correlations between IP scores and P3 peak amplitude, panel B between IP scores and P3 peak latency. Circles display electrodes, where correlations were significant.

To investigate the relation between cognitive performance and ERP indicators of brain activity, we correlated performance scores in the IP test, an indicator of perceptual speed, with P3 peak amplitude and peak latency as electrophysiological indicators of cognitive speed in young and older adults. The corresponding correlation coefficient distributions data for young adults showing significant correlations are topographically represented in Figure 7. As expected, P3 peak amplitude correlated positively with IP scores, at fronto-central, temporal and parietal sites, whereas P3 peak latency correlated negatively with IP scores, especially at fronto-central sites.

## Discussion

The main goal of this paper was to examine age-related changes in ERP components during Attend and Unattend conditions in the auditory oddball task. The principal finding from this study is that ERP components and corresponding difference waves change in peak amplitude, peak latency, and topological distribution across the lifespan. Results with respect to each of these three aspects are discussed in detail below.

### Lifespan age differences in primary ERP components

All ERP components except N1 showed significant main effect of age for peak amplitude. The P2 amplitude was higher in adults compared with children, whereas N2 showed the opposite effect. These differences were more pronounced at the frontal and central sites. Regarding age-related differences in the P2 amplitude in the literature, a linear increase of the amplitude was found with child age [8,37] and through adulthood [11]. In another study, P2 peak amplitude increases from 20 to 60 years and decreases thereafter [10]. In contrast, N2 peak amplitude decreased significantly from childhood to adulthood and old age [8,10,13,37]. In our study, children showed lower P2 amplitude than adults, which is apparently related to the higher N2 amplitude in children than in adults. The N2 component is responsible for the classification or categorization of deviant stimuli [17]. This categorization process seems to be very important for children, whereas among adults, at least a part of this process is apparently activated or passed as early as during the N1 and P2 components. Further evidence of the importance of the N2 component for children is that this component is also strongly developed for attended standard as well as for both deviant and standard stimulus under Unattend conditions, whereas in adults it is markedly reduced or failed. Interestingly, the development of the P2 wave in children seems to be "interrupted" by the rapid succession of the N2 wave, especially frontally. In contrast, the N2 component in adults is reduced because of the rapid succession of the P3 wave. On the other hand, these components (N1, P2, and N2) did not show significant age-related differences with respect to peak latency. The exception is a significant Age by Antero-Posterior interaction for the P2 latency, showing frontal-site prolonged latency in older adults and shorter latency in younger children. In other words, the differences observed in P2/N2 peak amplitudes could only be explained in dynamic terms, through the speed of potential changes or its acceleration and deceleration.

The later components, P3 and N3, showed significant age differences both in peak amplitude and peak latency. P3 peak amplitude was higher in older children and younger adults than in older adults, and N3 amplitude was higher in older children compared with both of the adult groups. In addition, both P3 and N3 peak latencies were significantly prolonged in older compared with younger adults. N3 latency was also shorter in younger adults than in older children. Similarly, other studies have found a significant decrease in P3 and N3 peak amplitude and an increase in P3 and N3 peak latency with advancing adult age [11,13,14,41]. As for age-related differences in the P3 component during childhood, extant findings are inconsistent (s. Table 1 for details). Some studies showed significant decrease in the P3 amplitude [39,43], whereas other studies showed significant increase in the P3 amplitude from childhood to adulthood [8,37]. In the present study, not only younger adults but also older children displayed significantly higher P3 amplitude than older adults, indicating that the P3 pattern in older children is similar to that in young adults. Although we did not find significant differences between younger and older children, it could be seen that P3 amplitude approaches the adult level only at age of 11–12 years. As mentioned above, the P3 component is known to be associated with context updating, orientation, processing termination, decision-making, and attentional or brain energy resource allocation when working memory is engaged (e.g., [1,18-20]. These processes are activated especially under the Attend deviant condition in children and younger adults and decreased markedly in older adults, as well as in all age groups under other conditions with restricted attentional load. Unfortunately, little is known about age-related changes of the N3 component in the literature. The significant decrease in N3 amplitude with advancing adult age compared with children is in accordance with the expectation [11,14,43] and may be due to synaptic density changes in the frontal cortex noted by Courchesne [44]. Because these two later ERP components, e.g., P3 and N3, are strongly linked to attentional processes during calculation of deviant stimuli and are markedly reduced under other conditions, it could be suggested that these components reflect the more non-automatic, controlled part of the stimulus processing. These processes became slower with advancing adult age.

In addition, practically all ERP components (also N1) showed significant Age by Antero-Posterior and Age by Laterality interactions, indicating age-related differences in the topological distribution of brain potentials. As mentioned in the result section, these differences are continuous across ages with a different direction for positive (moving from posterior to anterior with advancing age) and negative (moving from anterior to posterior with advancing age) ERP waves. Related to the lateral axis, topological distribution in children is more focused at central sites and spreads in adults (especially in older adults) to lateral sites (s. for details Fig. 3). It should be noted that although N1 peak amplitude did not show significant main effect of age, there were significant interactions between age and other factors (s. Table 2), suggesting that N1 is differentially pronounced in different brain regions and in different age groups. The strongest age differences were seen in the frontal regions, where older children showed the strongest N1 peak amplitude compared with other age groups, especially younger children and older adults (s. Fig. 3). This result confirms the assumptions that the maturation of the frontal N1 is not yet completed at the age of 9–12 years [25], and senescence-related changes for this component can be found in frontal regions (see [13]). In addition, other components except P3 (e.g., P2, N2, N3) showed most prominent changes with age at the frontal and central sites. These changes in topological distribution with advancing age are likely to be related to maturational and senescent changes that are particularly pronounced in frontal regions [74-77].

### Lifespan differences in difference-wave ERP components for change detection

The difference-wave ERP components presented in Fig. 4 indicating stimulus change detection (e.g., MMN and LDN) were most pronounced in the Attend condition. Statistical analyses of data obtained in this condition showed a significant main effect of age for peak amplitude and peak latency for LDN and a significant age effect for MMN peak latency. A post hoc test for LDN amplitude showed that it was higher in children, especially in older children, compared with adults. This result is in accordance with the literature [62,63] indicating enhanced neural activity for stimulus change detection, which is higher in children than in adults. These differences were more pronounced at the frontal sites, reflecting a higher activation of the frontal cortex during the stimulus change processing in children than in adults and indicating developmental changes of these mechanisms. In contrast to previous studies [25,62,63], our results show a clear dissociation between the earlier frontal component and the later parietal LDN component. This dissociation, which was clearer in younger and older adults, showed in children, especially in younger children, a very strong frontal component but a reduced parietal component overlapping with frontal activity. Since LDN is a late negativity peaking at about 600–800 ms, we suppose that in contrast to MMN, which indicates an automatic neural-mismatch process triggered by the sensory input from a rare deviant stimulus in the presence of a neural trace of the frequent standard stimulus [45,46], these mechanisms are non-automatic or controlled processes that are probably activated through task manipulation or counting of deviant stimuli. An additional confirmation for this is the fact that LDN in the Unattend condition was considerably reduced in children, especially OC, and was practically absent in adults. Furthermore, in the Attend condition there was a switch between the two processes, which were activated frontally early in the process and moved later to the parietal sites. This switching-mechanism is apparently not yet completed at the age of 9–12 years. Children showed LDN also at parietal sites, which is actually stronger than in adults, but these waves were overlapping with frontal waves, although OC showed switching to parietal activity dominance with much later onset latency. Often LDN (also called reorienting negativity, RON) is understood as reorienting, refocusing, or reallocation of attention back to the primary task ([25-27]). This functionality of RON is related to the stimulus (mostly, novel or distracter stimulus in a three-stimulus paradigm) eliciting this potential shift or response. The question about the functional meaning of the late negative wave was also discussed by Näätänen and colleagues [48] who suggested that " [i]t may represent a process which continues when a stimulus change is subliminal and so does not trigger endogenous processes. One important biological function might be to sensitise the organism so that a subliminal stimulus might be detected if repeated in close succession. This negativity might also reflect sensitisation processes subserving detection of the initial stimulus itself" (p. 93). This meaning is consistent with the suggestion that N3 (and accordingly LDN) can be superimposed by the CNV (Contingent Negative Variation) reflecting stimulus anticipation or expectancy [16,29]. Our results of high LDN only in the Attend condition and reduced or even absent LDN in the Unattend condition suggests that this component is modulated by active stimulus detection and is more likely related to counting of deviant stimuli. It is well known that besides the frontal lobe, the human parietal cortex, particularly the intraparietal sulcus (IPS), is implicated in processing generic numerical information or symbolic counting (s. for review [80,81]). Thus, both activity patterns (frontal and parietal) reflected in the two LDN subcomponents can represent serial counting processors. Further studies are needed to confirm these suggestions.

The latency of MMN was prolonged in OC compared with both of the adult age groups, and the latency of LDN was prolonged in OC and OA compared with YA. OC showed a slowing of both components, automatic and non-automatic, indicating general slowing (at least, compared with young adults) in the differentiation processes between deviant and standard stimuli. In contrast, OA were slowed only in the non-automatic processes indicating that cognitive slowing or cognitive decline during normal aging primarily affects these non-automatic processes, whereas the automatic processes seemed to be maintained. It may be that this process slowing is associated with decision-making or enhanced working memory, which are in the perfected or optimal form in younger adults. The fact that a significant slowing was not found in younger children may be due to the different functional meaning of these components, which showed also different topological distribution compared with other groups: the MMN showed temporal maxima compared with fronto-central maxima in adults, and the parieto-occipital maxima of the LDN component are markedly reduced in YC compared with OC and adults.

In the literature, MMN and also LDN results are not consistent, especially in children. Some studies reported adult-like MMN in 10–12 year old children but mostly absent MMN in 6–8 year old children [25,82]. At the same time, several studies have shown significant age-associated differences in the amplitude and latency of MMN [51-55]. Some studies reported a slight MMN peak latency decrease during the school-age years [55,56] and greater MMN amplitude in school-age children than in adults [57]. Older adults have been found to have smaller MMN amplitude than young adults in some studies [52,59] while in other studies young and older adults displayed similar MMN amplitudes [60]. Our results showed only local decrease in the MMN amplitude in adults as compared with children and shorter MMN peak latencies in adults as compared with OC (s. Fig. 5 for details). We agree with some of the existing literature that MMN is a developmentally quite stable ERP component, although it undergoes some some lifespan changes. Compared with MMN, LDN showed greater developmental changes and seems to reflect an important stimulus-detection mechanism, which is not yet fully understood. This is of particular interest because as mentioned LDN besides MMN can be found in newborns and even in the fetal brain [50]. In other words, LDN (and also MMN) seem to represent a basic stimulus change detection mechanism, which appears very early in ontogenesis and displays further changes across the lifespan.

### Lifespan differences in difference-wave ERP components for selective attention

While MMN and LDN reflect processing of stimulus change, the other difference wave components, i.e., EPN and LPN, indicate differences in stimulus processing when stimuli are attended or unattended through counting or not counting deviants. These difference-wave ERP components presented in Fig. 6 are most pronounced for deviant stimulus as compared with standard stimulus. Statistical analyses for deviant stimulus showed a significant main effect of age for peak amplitude and peak latency (except EPN). The amplitude of both components was higher in children than in adults: EPN amplitude was highest in YC (in OC it was only higher than in OA) and LPN was highest in OC. The selective attention enhancement associated with the counting of deviant stimuli induced a higher negative difference wave in children compared with adults. This attention-linked activation increase was greater in children, above all frontally, compared with adults, indicating a crucial role of the frontal cortex for selective attention in stimulus processing. In older children, the later part of this activation (LPN) was enhanced also at parietal sites. As mentioned above, the early component is proposed to reflect the processing of the sensory stimulus features and the later component the further processing of the stimuli and rehearsal of the attentional trace [68,69]. However, similar to MMN and LDN, we suppose that the early PN component, i.e. EPN, is connected with earlier attentional load supporting automatic stimulus processing, and the later PN component, i.e., LPN, is responsible for attention donation in the non-automatic controlled stimulus processing. This attention enhancement in both automatic and non-automatic processing of the deviant stimulus is higher in children than in adults, with the difference that the former attention-related component is stronger in YC and the later attention-related component is stronger in OC. This is evidence that selective attention processes in children require higher processing costs compared with adults. As shown in Fig. 5 and 6, if the frontal component of LPN is relatively equal in YC and OC, then the parietal component became more pronounced in OC. Like LDN, LPN contains two components: an earlier frontal and later parietal, which are most dissociated in YA. LPN showed also significant latency increase in OA compared with OC and YA, indicating a slowing of the selective attention mechanisms with non-automatic stimulus processing in old age.

Our results contradict, to some extent, earlier findings [70] indicating a higher amplitude of the Nd component (earlier and later) in adults as compared with children (mean age 8.1 years). On the other hand, Gomes et al. [72] could not find any differences in Nd amplitude between children (9 and 12 years old) and adults, and Bartgis et al. [71] showed a significant increase in the Nd amplitude in children from 5 to 9 years. Our results confirm, on the one hand, the tendency of an increase in the PN amplitude in childhood and, on the other hand, the tendency of a decrease in adulthood and old age. Furthermore, YC showed stronger involvement of earlier PN, whereas OC reduced compared with YC their earlier PN and enhanced the later PN, especially at parietal sites. This evidence suggests that selective attention mechanisms are not only different in children and adults but undergo some reconstructions during adulthood. Although we did not statistically test age-related differences in PN that were related to standard stimuli, the waveforms represented in Fig. 6 showed not only the same tendency for age-related changes but also indicated stronger involvement of selective attention mechanisms in processing non-target stimuli in children as compared with adults.

It should be noted here that the difference waves for stimulus change detection and selective attention, as displayed in Fig. 4 for Attend condition and in Fig. 6 for deviant stimulus, are in some respects very similar but this similarity should not deceive. The change detection components (MMN and LDN) were determined by the subtraction of the attended standard from the attended deviant, and attentional components (EPN and LPN) were determined by the subtraction of the unattended deviant from the attended deviant. Thus, this similarity is connected with the similarity in the waveforms of the attended standard and the unattended deviant. However, as shown in Fig. 4 and Fig. 6, these components have some differences in topological distribution, which also changes across ages. Furthermore, the EPN in children, as compared with adults, is topologically more extended and has a much earlier latency onset, indicating that the difference between attended and unattended stimuli in children concerns all three earlier primary ERP components (i.e., N1, P2, and N2), whereas in adults it is related above all to the N2 wave, similar to MMN during stimulus detection. Significant interactions of factor Age with factors Antero-Posterior and Laterality indicate age-related differences in the topological distribution of difference wave components in these two axes. Further analyses are necessary to answer the question: which cortical mechanisms underlie these changes in the topological distribution across ages.

### ERP components as electrophysiological indicators of cognitive speed

Event-related brain potentials are sensitive measures of the temporal dynamics and the intensity of stimulus-locked electrocortical activity during information processing. In line with this contention, we found that higher P3 peak amplitude and shorter P3 peak latency were significantly related to individual differences in perceptual speed among younger adults. Older adults compared with young adults failed to show significant correlation between P300 peak amplitude (and peak latency) and IP scores. Similar result found Iragui et al. [83] for correlation between slowed P300 latency and the reaction time in an auditory oddball task. The finding of an increase in the P3 peak amplitude and decrease in the P3 peak latency correlated with a higher performance in IP task indicates that higher perceptual speed requires higher activation of involved cell assemblies and faster neural responding. Interestingly, these correlations reach their maximum at the central regions (s. Fig. 7), indicating strong involvement of sensory-motor and somatosensory cortices as well as some close by regions of the frontal, parietal and temporal lobe in stimulus processing and perceptual speed. Whereas correlation between IP and P3 peak latency concerns some electrodes in the central and right-temporal regions, which are responsible for motor response and accurate timing, correlations between IP and P3 peak amplitude concern broader brain areas around the sensory-motor and somatosensory cortices, indicating that other processes besides the motor response and accurate timing (e.g., stimulus discrimination, decision making, etc.) are possibly also reflected in the strength of the P3 peak amplitude. Further lifespan studies with sample sizes that are sufficiently large to permit the application of structural equation modeling techniques are required to evaluate how these relations change across the lifespan.

## Conclusion

The main goal of this study was to examine age differences in event-related brain potentials using the auditory oddball task. We found substantial differences between children and adults as well as between younger and older adults in different (primary and secondary) ERP components. The differences between YC and OC were less pronounced, with OC often falling between YC and YA. Our results also underscore that when examining age-related differences across the lifespan, not only the several relevant ERP components should be compared separately, age differences in the temporal dynamic patterns between them are also of importance. As discussed above, the observed age-related differences in P2/N2 components could only be understood in light of their dynamical interactions. Stimulus change detection as well as selective attention mechanisms as reflected by corresponding ERP difference waves showed specific developmental changes across the lifespan, which were more pronounced during the transition from childhood to adulthood. Furthermore, age-related differences were found not only for peak amplitude and peak latency but also for the topological distributions of brain potentials indicating possible differences in stimulus processing and its cortical representation during development and normal aging. In sum, the present findings suggest that patterns of event-related brain potentials are highly malleable within individuals and undergo profound reorganization from childhood to early adulthood and old age. It should be noted here that these changes over the lifespan relate only to the two-tone-pip paradigm used in the study and can not be generalized to other paradigms (e.g., three stimulus paradigms) or to other kinds of stimuli (e.g., speech signals).

## Methods

### Subjects

All participants were volunteers and were recruited through announcements on Saarland schools (Gymnasiums) and Saarland University. The older adults were either auditors at Saarland University, participants in other continuing education programs, or both. For participation in the study, all subjects were paid 7.5 Euro per hour. All the subjects were right-handed, had no reported history of head or neurological disorders, and were not on medication. Individuals with a score of 34 or less on the Digit Symbol Substitution (DSS) test of the Wechsler Adult Intelligence Scale (WAIS: [84]) were excluded from the study. Of the participating individuals, 5 younger children, 1 older child, and 1 older adult were excluded from data analysis because they reported numbers of odd stimuli in Attend conditions (see below) that deviated more than 3 digits in either direction from the correct number. Thus, the effective sample consisted of twenty-four younger children (YC, mean age = 9.9, SD = 0.6, age range = 9.0–10.8 years, 13 females), twenty-eight older children (OC, mean age = 12.0, SD = 0.6, age range = 11.0–12.8 years, 14 females), thirty-one younger adults (YA, mean age = 22.7, SD = 1.6, age range = 18.8–25.1 years, 14 females), and twenty-eight older adults (OA, mean age = 67.8, SD = 3.0, age range = 63.9–74.5 years, 14 females). Participants of all ages including children were able to sustain their attention for the entire duration of the experiment. There were no significant differences between the age groups in the reporting numbers of odd stimuli. The study has been approved by the ethics committee of Saarland University and has therefore been performed in accordance with the ethical standards laid down in the 1964 Declaration of Helsinki. All subjects volunteered for this experiment and gave their written informed consent prior to their inclusion in the study. In the case with children, the parents did give consent for their children to participate in the study. Details that might disclose the identity of the subjects under study were omitted.

### Procedure

The EEG measurement began with a 3-minute relaxation phase (1.5 minutes with closed and 1.5 minutes with open eyes). The subjects sat relaxed in a chair in the electrically shielded room. The relaxation phase was followed by the auditory oddball task. During the task, the subjects received two different tone pips: a frequent 1000 Hz tone as a standard stimulus and a rare 800 Hz tone as a deviant stimulus. The standard and deviant stimuli were presented binaurally (with a probability of 0.8 and 0.2 for standard and deviant, respectively) through a headphone at 70 dB SPL with a duration of 50 ms. The inter-stimulus interval ranged from 1200 to 1500 ms. There were two different experimental conditions: passive listening (unattended) and active counting (attended). In the first condition, the subjects merely listened to the tone pips without any response, whereas in the second condition, the subjects had to attend to stimuli and to count the deviant tones. After the session, they were asked to report their counting results. Each experimental condition contained 152 standard tones and 38 deviant tones presented in a random order. The order of the conditions was always the same, that is, the passive listening condition was followed by the active counting condition.

### Psychological assessment

The BASE (Berlin Aging Study; cf. [85]) cognitive test battery was used for psychological assessment. Identical Pictures (IP) test from this battery, reflecting perceptual speed, was selected for correlational analyses of relations with electrophysiological data. The materials and procedural details of the cognitive battery have been described elsewhere [28].

### EEG recordings

The electroencephalogram (EEG) was recorded from 58 Ag/AgCl electrodes using an elastic cap (Electrocap International) with a sampling rate of 500 Hz in a frequency band ranged between 0.1 and 70 Hz. The left mastoid was used as a reference and the right mastoid was recorded as an active channel. The electrodes were placed according to the international 10–10 system. Vertical and horizontal electrooculogram (EOG) was recorded for control of eye blinks and eye movements. The EEG recordings were corrected for eye movements using the Gratton and Coles algorithm, and blink artefacts were rejected based on gradient criterion, i.e., maximal allowed voltage step (50 μV), and difference criterion, i.e., maximal allowed absolute difference of two values in the segment (200 μV). The analyses were conducted if the total number of artefact-free trials per condition was 25 or more. The mean number of artefact-free trials for attended deviant stimuli was in YC: 36.5 (1.9), in OC: 36.9 (2.1), in YA: 36.7 (3.3), and in OA: 35.9 (3.9). For other conditions the number of artefact-free trials was very similar.

### EEG data reduction and analyses

Separate ERPs associated with standard and deviant stimuli under Attend and Unattend conditions were calculated for each subject at each electrode site over a 1076 ms epoch, using a 50 ms pre-stimulus baseline correction and band pass Butterworth zero-phase digital filtering (0.3–20 Hz). Each ERP component was labeled according to its polarity (positive or negative) and peak latency: N1, P2, N2, P3 and N3. Peak amplitude (the largest value in a particular time window) and peak latency (the time point of the peak amplitude) were calculated for attended deviant stimuli only. The components were measured in the following time windows: N1 (70–150 ms), P2 (150–200 ms), N2 (200–300 ms), P3 (300–450 ms) and N3 (450–900 ms). In addition, a difference wave for the Attend condition was calculated as a difference between ERPs to the deviant and standard stimuli. In this difference wave, two negative components were determined: MMN (150–300 ms) and LDN (380–900). Another difference wave was calculated as a difference between ERPs to the attended and unattended deviant stimuli. In this difference wave, two negative components were identified: early processing negativity (EPN: 100–300) and late processing negativity (LPN: 300–900). Peak amplitude and peak latency were calculated for all these difference waves.

### Statistics

Amplitude and latency of ERP and difference wave components were entered into a three-way repeated measures ANOVA (Analysis of Variance) with the between-subjects factor Age (four age groups: YC, OC, YA, and OA) and within-subjects factors Antero-Posterior (3 levels: frontal, central and parietal) and Laterality (5 levels: left, medium left, mid-sagittal, medium right, right). Thus, 15 electrodes of the standard 10–20 system (frontal: F7, F3, Fz, F4, F8; central: T7, C3, Cz, C4, T8; parietal: P7, P3, Pz, P4, P8) were used to test age differences in peak amplitude and peak latency. The two factors (Antero-Posterior and Laterality) and their interaction reflect changes in topological distribution of the dependent variables across two axes: anterior-posterior and lateral. In all ANOVAs, Greenhouse-Geisser epsilons (ε) were used for non-sphericity correction when necessary.

To assess the relationship between performance scores in cognitive tests (IP, DS and DL) and P3 peak amplitude and peak latency, Pearson product correlations were computed. In the results section we presented only correlations with IP scores, because the relationship between the IP scores and EEG measures was more clearly. Given that sample sizes were too small to warrant age comparisons of correlational patterns, this analysis was restricted to the group of young and old adults.

## Authors' contributions

VM, YB, TvO, S-CL and UL designed the study. YB recruited the participants. TvO programmed the oddball task. VM and YB acquired and analyzed the data. VM, SC-L and UL drafted the manuscript. YB and TvO helped to draft the manuscript. All authors read and approved the final version of the manuscript.
